# Cognitive Impairment in Chronic Obstructive Pulmonary Disease (COPD): Possible Utility of Marine Bioactive Compounds

**DOI:** 10.3390/md16090313

**Published:** 2018-09-04

**Authors:** Giulia Prinzi, Alessia Santoro, Palma Lamonaca, Vittorio Cardaci, Massimo Fini, Patrizia Russo

**Affiliations:** 1 Clinical and Molecular Epidemiology, IRCSS San Raffaele Pisana, Via di Valcannuta 247, I-00166 Rome, Italy; giulia.prinzi@sanraffaele.it (G.P.); alessiasantoro92@gmail.com (A.S.); palma.lamonaca@gmail.com (P.L.); 2 Unit of Pulmonary Rehabilitation, IRCCS San Raffaele Pisana, Via della Pisana 235, I-00163 Rome, Italy; vittorio.cardaci@sanraffaele.it; 3 Scientific Direction, IRCSS San Raffaele Pisana, Via di Valcannuta 247, I-00166 Rome, Italy; massimo.fini@sanraffaele.it

**Keywords:** COPD, cognitive impairment, management strategy, marine bioactive compound, systems approaches

## Abstract

Chronic obstructive pulmonary disease (COPD) is characterized by long-term airflow limitation. Early-onset COPD in non-smoker subjects is ≥60 years and in the elderly is often associated with different comorbidities. Cognitive impairment is one of the most common feature in patients with COPD, and is associated with COPD severity and comorbidities. Cognitive impairment in COPD enhances the assistance requirement in different aspects of daily living, treatment adherence, and effectual self-management.This review describes various bioactive compounds of natural marine sources that modulate different targets shared by both COPD and cognitive impairment and hypothesizes a possible link between these two syndromes.

## 1. Introduction

Chronic obstructive pulmonary disease (COPD) causes chronic airflow limitation, breathlessness, exercise intolerance, cough, difficulty with daily activities, infections, and (re)hospitalization [1]. COPD pharmacological therapies are merely symptomatic, and not effective in disease modification and survival [1]. COPD is a multisystem disease, with effects beyond the lung and is associated with symptom burden and prognosis [2]. Many people with COPD have multiple other disorders [2]. The incidence of COPD in the general population is still increasing, and with an ageing population, this number is expected to increase further [3]. COPD is more prevalent in subjects older than 65 years of age. The onset of the disease is complex; multiple causes beyond smoking contribute to the development of COPD, such as environmental exposure, age-related degenerative changes, and genetic factors [4]. Thus, in elderly people, COPD is the result of different continuous gene–gene (G × G) and gene–environment (G × E) interactions that happen in the course of the life of a single person. COPD occurring at an earlier age than expected may result from the interaction of inherited factors and environmental exposures [5]. A seminal prospective study, published on 2014, of individuals 70 years and older, associates COPD with an increased risk of a-MCI or NA-MCI (mild cognitive impairement (MCI); amnestic MCI (a-MCI), and nonamnestic MCI (NA-MCI)). Moreover, the greatest risk for a-MCI and NA-MCI is among individuals with a duration of COPD longer than 5 years [6]. All of these data bring to light the importance of COPD as a risk factor for a-MCI and NA-MCI, highlighting the need for early intervention, to prevent or delay MCI onset and/or progression.

## 2. Mild Cognitive Impairment

The concept of MCI is evolving overtime, from the first definition of Reisberg and collaborators in the late 1980s [7,8,9,10,11,12,13,14], who considered MCI as an intermediate stage between normal ageing and dementia, to the definition of the National Institute on Aging-Alzheimer’s Association (NIA-AA) working group in 2011, who proposed new criteria for MCI [14]. The NIA-AA assumes that the decline in multiple (cognitive) domains, and single non-memory domain subtypes, are greater than would be expected considering the patient’s age, gender, and educational background [14]. The NIA-AA suggests that biomarkers may be used in research settings to aid in the identification of MCI subtypes (i.e., MCI due to AD or MCI that is unlikely due to AD). MCI matches to mild neurocognitive disorder in the new Diagnostic and Statistical Manual of Mental Disorders, fifth edition, (DSM-5). DSM-5 is the taxonomic and diagnostic tool published by the American Psychiatric Association (APA) that, in the United States, serves as the principal authority for psychiatric diagnoses and neurological disorders. [9]. A differentiation between MCI with amnestic MCI (a-MCI) or without impairments in memory domain is assumed (NA-MCI, (e.g., executive control, language, or visuospatial abilities)). Moreover, whereas a-MCI is presumed to have a high risk of converting to AD, subjects with NA-MCI have a high risk of converting to non-Alzheimer’s dementia [10]. Longitudinal studies have shown that MCI patients may return to a non-MCI diagnosis after some years [11,12]. These findings may imply either that MCI is a high risk but transient phase, or that diagnosis and/or screening is still unfocused. Indeed, different genetic and environmental factors may contribute to MCI [13].

Actually determining the primary underlying etiology of MCI is still a challenge, and is strictly dependent to the patient’s history. Some neuroimaging, genetic, and neuropsychological assessment may be considered as suggested by NIA-AA (see Table 1) [8,14,15,16,17,18,19,20,21,22].

Currently, there are no accepted pharmacologic treatments for MCI approved by the FDA (U.S. Food and Drug Administration), the EMA (European Medicines Agency), or the Pharmaceuticals and Medical Devices Agency in Japan. Table 2 reports the effect of different drug approaches on MCI, according to the Agency for Healthcare Research and Quality (USA) [23].

None of the proposed interventions has indicated effectiveness and/or helpfulness in delaying the progression from MCI to AD dementia [46,47,48,49]. Importantly, as highlighted recently by Petersen et al. [49], there are no high-quality, long-term studies recognizing pharmacologic or dietary drugs able to improve cognition and/or delay progression in patients with MCI. Moreover, Petersen et al. [49] recommended that patients diagnosed with MCI should avoid acetylcholinesterase inhibitors (AChEIs; authorized drugs for AD, i.e., donepezil, rivastigmine, galantamine), since these drugs show no benefit on cognitive outcomes or reduction in progression from MCI to dementia, and side effects, including gastrointestinal symptoms and cardiac concerns.

Thus, on the light of the heterogeneity of MCI, further studies testing different compounds in all of its subtypes are necessary to draw clear conclusions.

### 2.1. Association of Chronic Obstructive Pulmonary Disease with Mild Cognitive Impairment and Dementia

Starting in 2011, important studies [6,50,51], using standardized *criteria*, found that patients with moderate to severe COPD are at high risk for MCI. The association between COPD and MCI remains significant even after adjusting for cardiovascular comorbidities and other covariates (i.e., hypoxemia, hypercapnia). A recent systematic review and meta-analysis of observational studies, including 23,116 subjects with COPD with a mean age of 66.3 years, found that the prevalence of MCI is 25% [52]. These findings are in agreement with the most recent observations of Ouellette and Lavoie [53]. The prevalence of MCI in the general population is 8.4% for people of 65–69 years [49]. These data may suggest that in the presence of severe COPD, the percentage of MCI is higher than in the general population. Until now, no explanation has been proposed for this association. The underlying mechanisms of cognitive impairments in COPD are debatable and poorly understood. It has been hypothesized that serum clusterin (CLU) plays a role. In severe COPD patients, the levels of serum CLU are very high, and the level of serum CLU is negatively correlated with cognitive ability [54]. The peripheral CLU concentration is associated with mini-mental state examination (MMSE) score and brain atrophy in both MCI and AD patients [55,56]. Moreover, the CLU levels are higher in MCI individuals who convert to dementia after one year, than to non-converters [57]. Secretory CLU, also known as apolipoprotein J (apoJ), is a stress-activated ATP-independent molecular chaperone. CLU is a highly glycosylated glycoprotein of 80 kDa, consisting of two polypeptide chains connected by four to five disulfide bonds. CLU/ApoJ is involved in different transcriptional networks controlling protein homeostasis/proteostasis, apoptosis/pro-survival signaling [58]. On the other hand, it has been reported that, in human lung fibroblasts exposed to cigarette smoke, there is a high accumulation of CLU/ApoJ protecting, presumably, lung fibroblasts against cigarette smoke-induced oxidative stress [59].

It seems that the existing relationships between COPD and MCI are independent of the presence of comorbidities (i.e., vascular risk factors and stroke) [6,51]. COPD is associated with premature aging characterized by chronic inflammatory process [36], which may have a role in the cognitive impairment. Indeed, COPD shows different hallmarks of aging [36,60] such as follows:

*i.* Abnormal microRNA pattern. MicroRNAs (miRNAs), a class of small non-coding RNAs, are involved in post-transcriptional gene repression. Alterations in miRNA abundance occurs in lung tissue, inflammatory cells, and freely circulating cells in blood, and are thought to function both as drivers and modifiers of disease [61,62]. In COPD patients, -miR-124-3p, miR-34a, miR-124, miR-29c and miR-126 are upregulated; -miR-181c, miR-21, miR-146a, miR-98-5bp are downregulated [61]. In MCI/AD, miRNAs (miR-124-3p, miR-34a, miR-124, miR-181c, miR-21, miR-146a, miR-98-5bp) contribute to the development, differentiation, and synaptic plasticity of neuronal cells, and are involved in many neurodegenerative diseases, including AD [62].
*ii.* Activation of PI3K-mTOR signaling. PI3K-AKT-mammalian target of rapamycin (mTOR) pathway is critical for cellular senescence and aging. In parallel, mTOR is a negative regulator of autophagia. There is evidence for PI3K activation in the lungs and cells of COPD patients, as shown by increased expression of the downstream kinase phosphorylated Akt, which in turn activates mTOR [36]. mTOR has a critical role during cognitive function and memory and affects genetic pathways that lead to cognitive loss. An mTOR upstream signaling pathway, the PI3K/Akt axis, is observed in AD brain. Persistent activation of neuronal mTOR signaling is found in MCI and AD brains [63].
*iii.* Altered autophagy. Refers to a pathway of cellular self-digestion controlling the degradation of subcellular constituents, including misfolded proteins and damaged organelles. Increasing numbers of autophagic vacuoles are observed in COPD lung tissues under electron microscopy, whereas low vacuole formation is observed in control tissues. Autophagy and mitophagy play a complex role in the lungs, and its related phenomena can have both protective and injurious effects on the progression of COPD. Currently, there is no unifying explanation for the discrepancies between various studies [64]. Although the exact pathological role of autophagy in AD remains to be elucidated, autophagy inducers might provide a new effective therapeutic strategy by degrading aggregates in the early stages of AD. By contrast, the activation of autophagy might enhance disease severity during the late stages of AD, by accelerating Aβ-amyloid production.The autophagy–lysosome pathway is unable to “keep up” with the misfolded protein load that is built up, and becomes defective, causing the aggregation of protein [65]. A mutation in sequestosome1 (SQSTM1), a marker for autophagy that binds cargoes, is identified in patients with familial AD [66].
*iv.* Decreased anti-aging molecules. Many endogenous antiaging molecules counteract the mechanisms of senescence, and a reduction in their expression may accelerate the aging process [36]. Sirtuins, are recognized as antiaging molecules that regulate lifespan. Sirtuins are highly conserved NAD^+^-dependent deacetylases enzymes that play a role in resistance to stress, genomic stability, and energy metabolism. Defective sirtuins are proposed as a mechanism for accelerated lung aging in COPD. SIRT1, 120 kDa (actual size), levels are decreased in patients with COPD, as a result of oxidative stress [67]. SIRT1 decreases with increasing severity of lung emphysema and with a clinical history of frequent COPD exacerbations. Over-expression of a miR-34a causes a significant reduction in both mRNA and protein of SIRT1/-6 in COPD. The aging-suppressor gene, *Klotho*, is downregulated in COPD [67].There is no direct proof of different levels of SIRT1 in human AD, but overexpression of miR-34a decreases SIRT1 levels. Klotho protein is predominantly secreted by the choroid plexus of the brain, and protects hippocampal neurons from amyloid and glutamate toxicity via the activation of an antioxidant enzymatic system, suggesting *Klotho* is necessary for oligodendrocyte maturation and myelin integrity.The *Klotho* KL-VS variant is associated with an increase in the incidence of dementia in older men, in a dose-dependent fashion [68].
*v.* Defective DNA damage repair. Effective repair of DNA damage is essential for the survival of cells, and most individual organisms and species. Ineffective repair can result in cell death, cancer, and neurological disease. COPD and AD are associated with excessive DNA damage [69,70]. Several types of DNA damage are associated with neurodegeneration, including bulky adducts, abasic sites, DNA single-strand breaks (SSBs), DNA double-strand breaks (DSBs), base mismatches, insertions, and deletions. DNA repair inefficiency is common in COPD, and is correlated to progression. Poly (ADP-ribose) polymerase (PARP) activation is associated with the progression of COPD [71]. BRCA1 (originally breast cancer 1; currently BRCA1) critically contributes to DSB repair in central neurons and neuronal reductions [69]. BRCA1 causes increased persistence of DSBs, abnormal chromatin remodeling, cellular dysfunction, and cognitive deficits. Depletion of BRCA1 is found in brains of patients with MCI or AD. Depletion of BRCA1 is caused by the pathological accumulation of Aβ, which may promote the proteasomal degradation of BRCA1 through overactivation of extrasynaptic *N*-methyl-d-aspartate (NMDA) receptor [69].
*vi.* Cellular senescence. Senescence is a state of irreversible cell cycle arrest. Senescent cells accumulate in the lung of COPD patients leading to persistent secretory phenotype (SASP) factors, and contributing to increased tissue dysfunction and COPD severity [72]. p16 and p21 are upregulated in cells of COPD patients. Compared with controls, p21 level is significantly decreased in lymphocytes of AD patients, while p53 is increased [72].
*vii.* Epigenetic changes. They include DNA methylation, covalent modifications of histone proteins and non-coding RNAs, and increases or decreases in gene transcription. In patients with COPD, there is emerging evidence showing aberrant expression of epigenetic marks, such as DNA methylation, histone modifications, and microRNAs in blood, sputum, and lung tissue [73]. AD entails dramatic losses of histone H4 lysine 16 acetylated isoform (H4K16ac) in the proximity of genes linked to aging and AD. A targeted proteomics approach in human brains showed reduction of histone H3 lysine18 acetylation (H3K18ac) and histone H3 lysine 23 acetylation (H3K23ac) in AD [74].
*viii.* Immunosenescence. Immunosenescence affects both innate and adaptive immunity, leading to a loss of function, and is implicated in chronic inflammatory diseases. In COPD, there is reduced immunity, which results in a low-grade chronic inflammatory response. Cluster of differentiation 8 + 28 (CD8 + CD28) null cells from COPD patients have reduced histone deacetylase 2 (HDAC2) expression and are corticosteroid resistant [36]. Patients with mild AD show decreased percentages of naïve cells, elevated memory cells, and increased proportions of CD4^+^, but not CD8^+^ cells lacking the important costimulatory receptor CD28 [75].
*ix.* Mitochondrial dysfunction. The key function of mitochondria is to produce ATP through the coupling of oxidative phosphorylation with cellular respiration. Mitochondria play a crucial role in the maintenance of intracellular Ca^2+^ homeostasis, because they can take up substantial amounts of cytosolic Ca^2+^. COPD is linked to increased mitochondrial reactive oxygen species (ROS) production, decreased intracellular antioxidants, and reduced numbers of mitochondria. The mitochondrial stress markers Parkin and PTEN-induced protein kinase-1 (PINK1) are increased in COPD patients [36]. Mitochondrial deficiency has been suggested to be a hallmark of AD as the patients display early metabolic changes prior to the emergence of any histopathological or clinical abnormalities, showing reduced metabolism, disruption of Ca^2+^ homeostasis, increased levels of ROS, lipid peroxidation and apoptosis, as well as hyperphosphorylation, aggregation, and damage of axonal transport leading to abnormal mitochondrial distribution. These pathological features impair mitochondrial dynamics by regulating mitochondrial fission/fusion proteins, causing mitochondrial dysfunction and neuronal damage [76].
*x.* Stem cell exhaustion. Airway basal progenitor cells are crucial for lung health and resilience because of their ability to repair injured airways. Basal progenitor count, self-renewal, and multipotentiality are all reduced. COPD progenitors produce an epithelium with increased basal and mucous cells and decreased ciliated cells, replicating the COPD phenotype [77].
*xi.* Telomere shortening. Studies analyzing leukocyte telomere length (LTL) at the population level have provided ample evidence for the hypothesis that LTL shortening is associated with aging and with age-related chronic diseases (cardiovascular and metabolic disease, cancer), although some inconsistencies have been observed.The exact mechanisms leading to telomere shortening in association with COPD are not yet understood [36]. Increased oxidative stress impairs telomerase activity and, thus, may directly result in telomere shortening. Telomere shortening, in turn, leads to the activation of p21, resulting in cellular senescence and the release of proinflammatory mediators, such as interleukin (IL)-6 and chemokine CXCL8. The telomere length and its rate of shortening did not relate to clinical and lung function parameters [36]. Intermediate values in the aMCI subjects, and the lowest values in the AD patients, suggest a definite relationship between telomere reduction and AD development. Association between APOE genotypes and LTL is observed [78].

All of these data are useful for finding new molecules able to act both in COPD and in MCI/AD.

### 2.2. Marine Bioactive Compounds and COPD and MCI/AD

Today, ocean habitats are the newest frontier in drug medical research. Thus, starting in 1969, FDA/EMA approved eight drugs obtained by marine sources including important antineoplastic (cytarabine, eribulin, trabectidin) and anti-pain (ziconotide) compounds [79]. The actualization of this relatively new area of scientific exploration is based on continuous testimony of marine medicine that comes from 2953 BC during emperor Fu His in China, as a tax for profits of fish-derived medicine [80]. Actually, we published several reviews on this topic [81,82,83,84]. In one, we described the most promising agents from marine sources in the treatment of AD [84]. Here, Table 3 reports new marine bioactive compounds acting against different targets shared by COPD and MCI/AD.

Drug activities and drug mechanisms of action are investigated on cellular and/or in mouse model systems. Every marine bioactive compound is studied in a single disease (COPD or MCI/AD) model [66,85,86,87,88,89,90,91,92,93,94,95].

**Figure 1:**
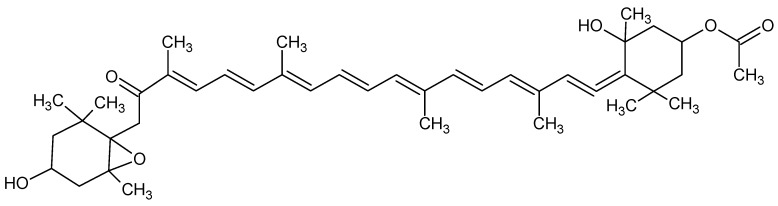
Fucoxanthin.

Fucoxanthin (Fx) is a xanthophyll, with chemical formula C_42_H_58_O_6_ (Figure 1), isolated from edible brown seaweeds firstly from *Fucus*, *Dictyota*, and *Laminaria*. In lung an inhibition of TGF-β1-induced phosphorylation of p38 mitogen-activated protein kinase (MAPK), phosphatidylinositol 3-kinase (PI3K)/Akt, and Smad2/Smad3 (Smad2/3) after Fx treatment, has been observed. Collagen contraction decreased significantly upon Fx treatment. Intraperitoneal injection of Fx in mice inhibits bleomicyn-induced lung fibrosis [85]. Fx attenuates Aβ oligomer-induced neurotoxicity on SH-SY5Y cells (isolated from a bone marrow biopsy of a neuroblastoma used as “in vitro models” of neuronal function and differentiation) leading neuroprotective effects via regulating PI3K/Akt and ERK pathways [86].

**Figure 2:**
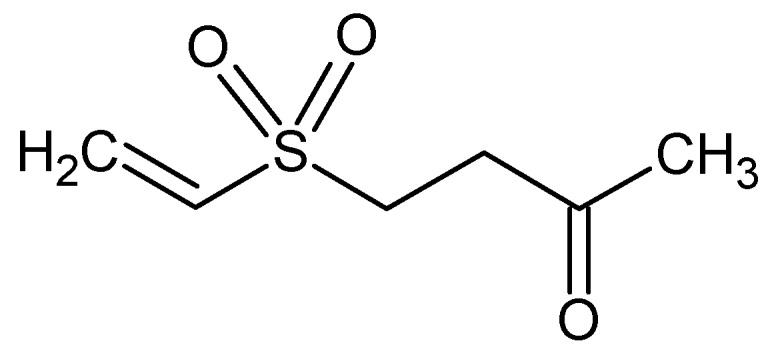
Austrasulfone.

Austrasulfone, with chemical formula C_6_H_10_O_3_S (Figure 2), from the soft coral *Cladiella australis*, collected in Taiwan waters, shows anti-apoptotic activity on neuronal cells SH-SY5Y mediated through the regulation of the Akt and heme oxygenase (HO)-1 signaling pathways [87].

**Figure 3:**
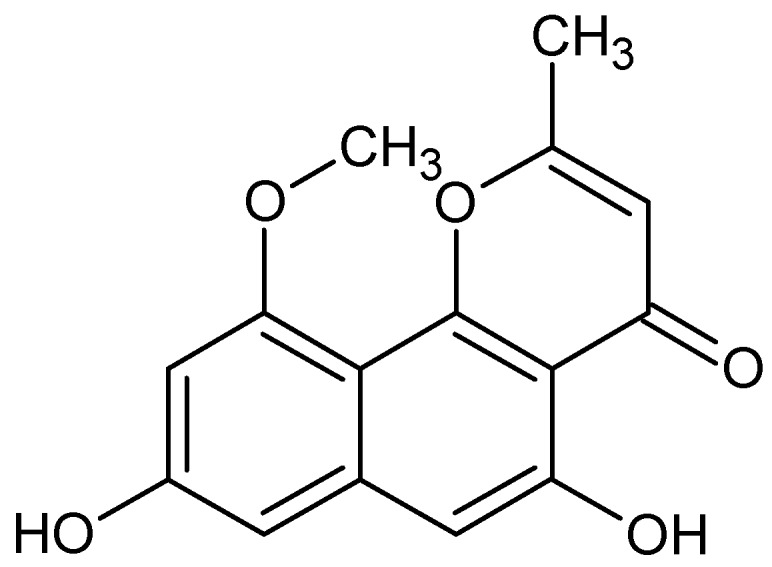
TMC-256C1.

TMC-256C1, with chemical formula C_15_H_12_O_5_ (Figure 3), isolated from an ethyl acetate extract of the marine-derived fungus *Aspergillus sp. SF6354*, activates p38 mitogen-activated protein kinases (MAPK) and PI3K/Akt signaling pathways in mouse BV2 microglial cells [88].

**Figure 4:**
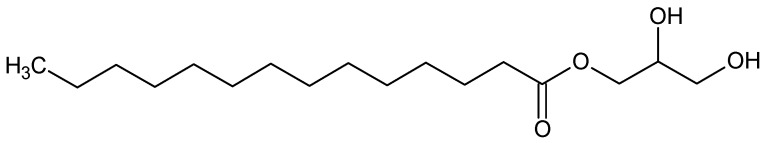
1-*O*-(Myristoyl) glycerol.

1-*O*-(Myristoyl) glycerol (MG), with chemical formula C_17_H_34_O_4_ (Figure 4), from the head of the fish *Ilishaelongate*, induces 42% of the neurite outgrowth of rat PC12 (rat adrenal gland pheochromocytoma) cells through the activation of ERK, cAMP responsive element-binding protein (CREB) and PI3K signaling pathways [89].

**Figure 5:**
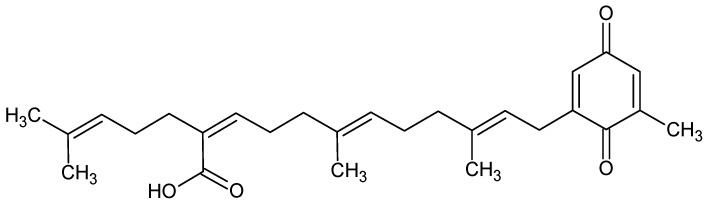
Sargaquinoic acid.

Sargaquinoic acid, with chemical formula C_27_H_36_O_4_ (Figure 5), from a marine brown alga *Sargassum macrocarpum*, enhances neuritere generation and protected rat PC12D cells from hydrogen peroxide-induced oxidative stress through PI3K signaling pathways [90].

**Figure 6:**
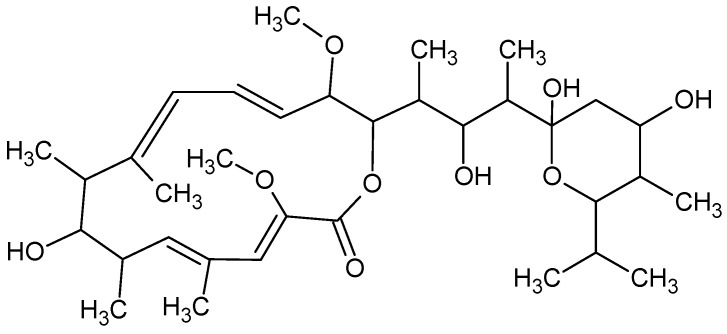
Bafilomycin.

Bafilomycin(s), with chemical formula C_35_H_58_O_9_ (Figure 6), a family of toxic macrolide antibiotics from marine *Streptomyces griseus*, inhibits autophagy by preventing fusion of autophagosomes with lysosomes [66].

**Figure 7:**
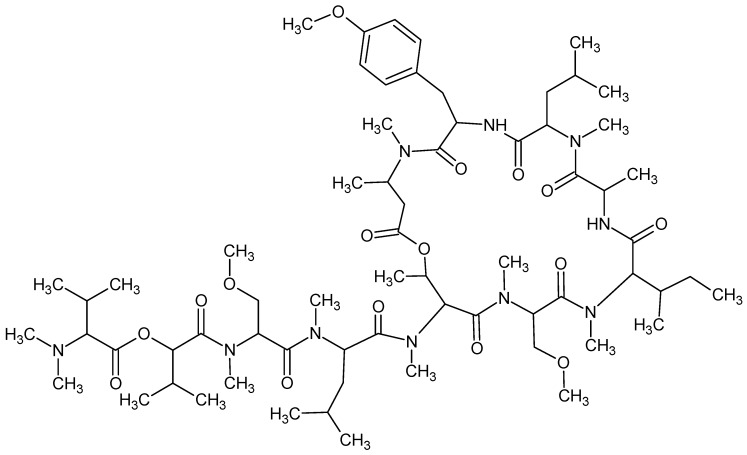
Coibamide A.

Coibamide A, with chemical formula C_65_H_110_N_10_O_16_ (Figure 7), an antiproliferative depsipeptide isolated from a marine *Leptolyngbya* cyanobacterium, induces autophagy in an autophagy-related gene 5 (*Atg5*)-dependent manner [66].

**Figure 8:**
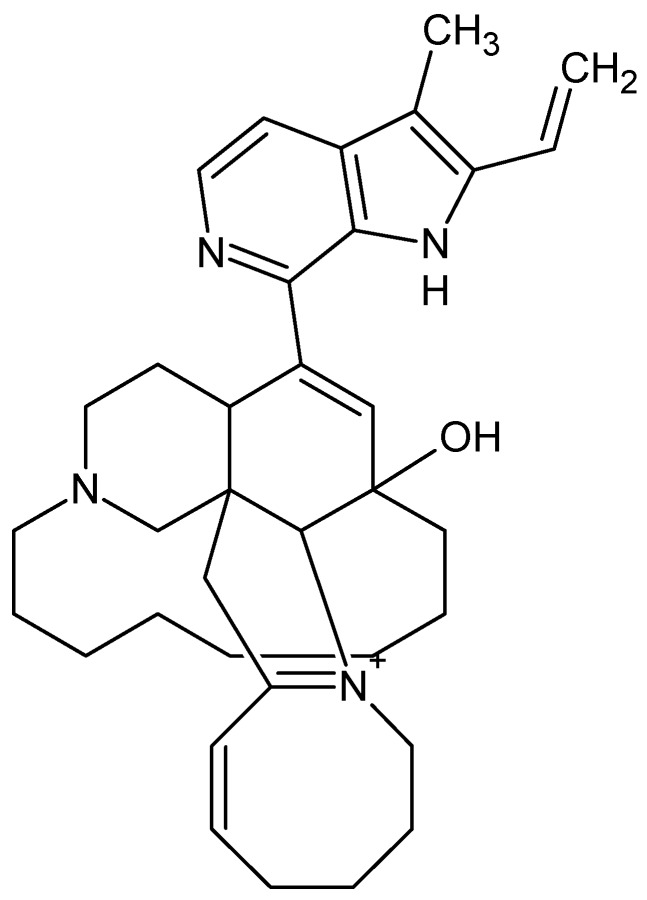
Manzamine A.

**Figure 9:**
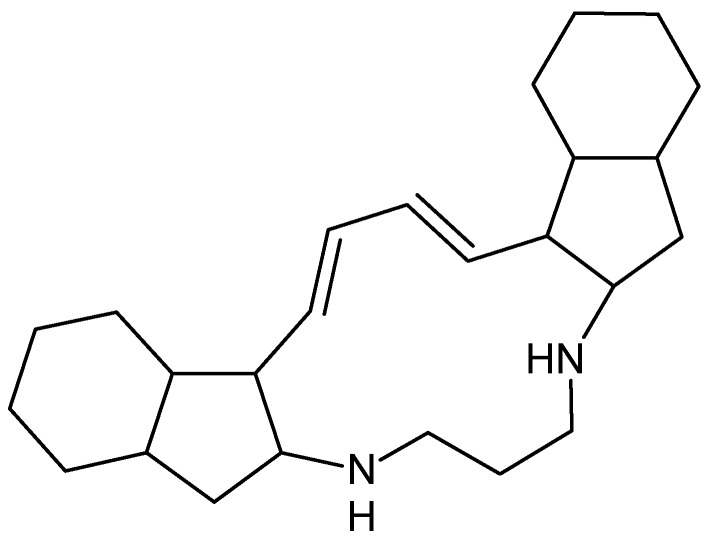
Papuamine.

Manzamine A, with chemical formula **C**_36_H_44_N_4_O (Figure 8), a β-carboline alkaloid isolated from the marine sponge *Xestospongiaashmorica* that acts as a GSK-3β inhibitor and papuamine, with chemical formula C_25_H_40_N_2_ (Figure 9), a pentacyclic alkaloid and antifungal agent, fromthe marine sponge *Halicona*, induces autophagy by increasing levels of microtubule-associated protein 1 light chain 3 (LC3) [66].

**Figure 10:**
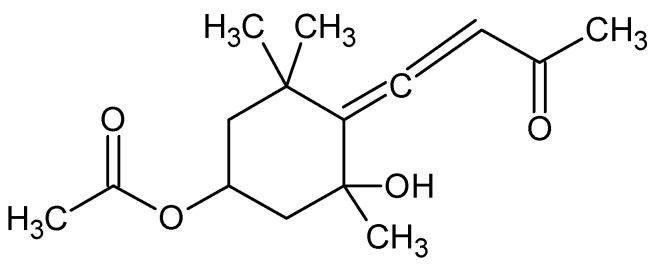
Apo-9′-fucoxanthinone.

Apo-9′-fucoxanthinone (ApoF9), with chemical formula C_15_H_22_O_4_ (Figure 10), from brown algae *Undariopsis peteseniana*, decreases cigarette smoke extract-induced DNA damage in immortalized human bronchial epithelial cells via reduction of ATM (atypical kinase of the PIKK family) phosphorylation [91].

**Figure 11:**
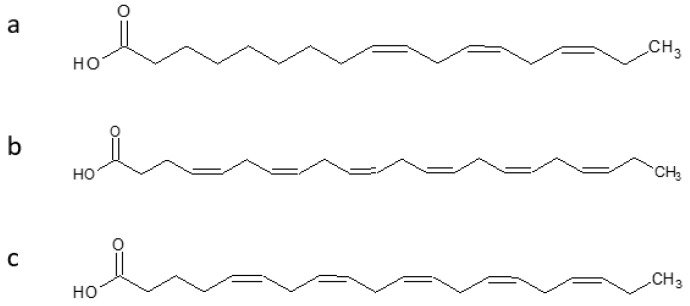
Omega-3 fatty acids. (**a**) Alpha-linolenic acid. (**b**) Docosahexaenoic acid. (**c**) Eicosapentaenoic acid.

**Figure 12:**
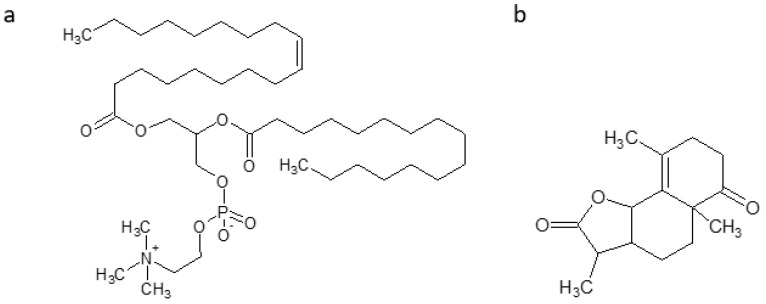
Phosphatidylcholine (**a**) and gracilin (**b**).

Antarctic krill oil (AKO), from *Euphausia superb*, is rich in polyunsaturated fatty acids, and two of the most important components are omega-3 fatty acids similar to those in fish oil (Alpha-linolenic acid (ALA) with chemical formula C_18_H_30_O_2_ (Figure 11a), docosahexaenoic acid (DHA)with chemical formula C_22_H_32_O_2_ (Figure 11b), eicosapentaenoic acid (EPA) with chemical formula C_20_H_30_O_2_ (Figure 11c)) and phospholipid-derived fatty acids (PLFA), mainly phosphatidylcholine with chemical formula C_46_H_84_NO_8_P (Figure 12). A protective effect was observed against AD in senescence-accelerated prone mouse strain8 (SAMP8). AKO ameliorates learning and memory deficits, and eases the anxiety by Morris water maze, Barnes maze, and open-field test. AKO reduces Aβ accumulation in hippocampus by decreasing the contents of malondialdehyde and 7,8-dihydro-8-oxoguanine, by increasing superoxide dismutase and glutathione peroxidase activities in the brain of SAMP8 mice [92].

A 43 kD protein, isolated from atlantic oysters, attenuates neuronal cell death induced by 100 mM d-galactose on human neurons-hippocampal (HN-h) cells in a dose-dependent manner. This protein alleviates mitochondrial inactivation, decreasing mitochondrial membrane potential oxidative stress, and fusion/fission state at non-cytotoxic concentrations of d-galactose-treated HN-h cells. The induced recovery of metallathionein-3 (MT-3) decreases and inhibits β- and γ-secretase, as well as Aβ accumulation in HN-h cells caused by d-galactose induction [93].

Gracilin(s), with chemical formula C_15_H_20_O_3_ (Figure 12), a family of diterpenoid compounds, isolated from the sponge *Spongionella*, in 3xTg-AD mice (triple-transgenic mouse model of AD, the only model that exhibits both Aand pathology characteristic of the human form), after chronicintraperitoneal treatments, in preliminary behavioral test, points to a positive trend on learning and spatial memory of treated mice. Gracilins decrease Aβ_42_ and hyperphosphorylated levels, inhibit ERK and β-secretase enzyme1 (BACE1),and preserves neurons against oxidative damage [94,95].

## 3. Discussion

The majority of new marine bioactive compounds are investigated in the field of neurodegeneration. COPD appears to be a neglected field of research. Nevertheless, according to the US Burden of Disease Collaborators, in 2016 COPD was the third cause of death in the United States, with an increasing trend in comparison with 2010 [96]. In Italy, COPD affects 3.5 million people, accounting for 55% of deaths/year among respiratory disease (3rd cause of death) [97]. COPD causes chronic airflow limitation, breathlessness, exercise intolerance, cough, difficulty with daily activities, infections, and (re)hospitalization [1]. COPD pharmacological therapies are merely symptomatic, and not effective in diseasemodification and survival [98]. Patients experience exacerbations, which contribute to high rates of emergency department (ED) visits, and in-patient admissions and readmissions, and high costs to the economy [99].The pattern of care for people with moderate–very severe COPD involves regular lengthy hospital admissions and rehospitalization for acute exacerbations (AECOPD), which result in high healthcare costs (Italy: 2723€/year/patient; 2617€/patient for ED) [99]. Costs increase with disease severity, presence of comorbidity, and rehabilitation [99], with an undesirable effect on the quality of life patient self-management. AECOPD accounts for 0.5% of ED visits, and are economically onerous [99]. Yet, most healthcare resources are poured into managing acute exacerbations that are only temporarily effective. AECOPD patients attending ED are old, affected by several comorbidities, and are burdened by a high prevalence of adverse outcome [99].

The first report of an association between COPD and AD is recordedin 1982 [100]. Since then, different studies, reporting this association, observed that either COPD worsens AD and increases the rate of MCI [6,50,51,53,54] or that AD worsens the severity of COPD [101,102,103]. This is a “vicious circle” that makes (self)-management difficult [103] especially for persons with neurocognitive impairments. Self-management in COPDimplies the personal ability to monitor symptoms, adhere to therapy, sustain an healthy lifestyle, and cope with the impact of diseases on daily functioning, emotions, and relationships [103]. There is contrasting evidence as to whether cognitive impairment (based on MMSE scores <24) is an independent predictor of poor-quality of spirometry [104,105]. Spirometry, a measure of forced expiratory volume in 1 s (FEV1), is a gold standard for the diagnosis and assessment of severity and follow-up of COPD [1].

Although, the present gold-standardrecommendations recognize comorbidities as important to COPD prognosis and severity, in general, they suggest that the presence of comorbidities should not alter COPD treatment, and “should be treated per usual standards regardless of the presence of COPD” [1]. This approach reflects the concept of “comorbidity” or “multi-morbidity” as a simple sum of single diseases. Actually, this concept appears misleading, possibly reflecting an oversimplification. It is well known now that a disease phenotype, the presentation of a disease in a given individual, reflects various pathobiological processes that interact in a complex network, involving genome, transcriptome, proteome, metabolome, and environment. The coexistence of two or more diseases in the same person raises the question of the presence of a possible common etiological pathway determined either by common disease genes or by a co-regulated cellular pathway, the so called “shared component hypothesis”. The shared component model, as proposed by Knorr-Held andBest [106], represents an easy approach to the joint spatial analysis of two related diseases. The key idea of the shared component model is to separate the underlying risk surface for each disease into a shared and a disease-specific component. At the molecular level, a disease may be considered as a disorder of a network of interactions, difficult to be considered as being independent of one to another [107,108]. Consecutively, a drug acting on a single target results in a phenotypic reaction caused by changes in the multi-dimensional networks, rather than exclusively in the target action. The network may be involved in *(i)* a unique disease pathway or *(ii)* belonging to multiple disease pathways. In this *scenario*,precision pharmacology (PP), a new theoretical paradigm, has in mind to help in exploring andpredicting the whole effect of a given drug at the systems level (molecular, cellular, tissue, organ, organism, and population levels) [109].The long-term goals of PP are both *(i)* developing multi-target therapeutic approaches for multifactorial diseases, such as COPD and AD; and *(ii)* finding frameworks of therapeutic efficacy/adverse risks [108,110].

Marine bioactive compounds, by nature, are often structurally complex compounds with efficacy relying on multitarget intervention via their multiple active components. Thus, they can be examined for developing network-based multicomponent drugs. The example of bryostatin (Bry-1), a potent modulator of PKC, initially isolated from the extract of *Bugulaneritina,* is emblematic of marine bioactive compounds’ potential, since Bry-1 is exploited in, according to the classic concept, different non-correlated diseases, such as cancer, HIV, and neurodegenerative diseases [84].

In Table 3, it appears that similar patterns are involved both in COPD and in MCI/AD. The same drug, such as fucoxanthin, is useful both against lung fibrosis and neurodegeneration [85,86]. Moreover, Fx may reverse scopolamine-induced impairments of cognition in mice, increasing choline acetyltransferase (ChAT) activity and brain-derived neurotrophic factor (BDNF) expression, and decreasing AChE activity in the brains. Moreover, Fx directly inhibits AChE in a non-competitive manner in vitro, possibly interacting with the peripheral anionic site within AChE [111]. In scopolamine-induced cognitive impairments, oxidative stress plays an important role, thus, it may be possible to speculate that Fx reverses impairments of cognition, reducing the oxidative stress increase. Patients affected by COPD and MCI/AD may benefit from Fx administration. However, additional experiments to address this shall be planned.

A recent paper revised the literature for the selection of cancer genes for the multitargeted use of existing drugs and natural products [112]. The authors used network analysis and a search tool for retrieval of interacting genes/proteins (STRING), to study the possible interactions to show the links between antioxidants, antibiotics, anti-inflammatory and antimitotic agents and their targets, for their possible use in cancer. After network analysis, they obtained a shortlist of 22 genes based on their average shortest path length, connecting one node to all other nodes in a network [112]. The selected genes were then analyzed with STRING for their protein–protein interactions. Accordingly, the authors proposed the selected genes to be regarded as main targets, and used in finding marine bioactive compounds as drug leads in cancer treatment.

We hereby suggest a screening of marine bioactive compounds having antioxidant, anti-inflammatory, anti-mitochondrial dysfunction, and anti-accelerated aging activity on COPD and MCI/AD models.

## Figures and Tables

**Table 1:** Biomarkers to assess mild cognitive impairement (MCI) according to National Institute on Aging-Alzheimer’s Association (NIA-AA) [14].

Markers	Methodology	References
**AD PATHOLOGY**		
Biomarkers of amyloid (Aβ) deposition	-Cerebrospinal fluid (CSF) concentrations of Aβ_42_ (decreased CSF Aβ_42_ levels) -Positron emission tomography (PET) amyloid imaging	[14,15]
Biomarkers of neuronal injury	-CSF concentrations of /phosphorylated(increased CSF/p levels) -Hippocampal volume or medial temporal atrophy or rate of brain atrophy measured by structural MRI -Decreased glucose metabolism in temporoparietal regions on fluorodeoxyglucose PET imaging	[15,16,17]
Presence of AD genetic risk factors	Variation in apolipoprotein E gene located on chromosome 19	[18,19]
**LEWY BODY PATHOLOGY**		
-Visual hallucinations -Parkinsonism -Motor features (bradykinesia, rigidity) -Rapid eye movement during sleep -Abnormalities suggesting pathological processes associated with dementia and with Lewy bodies (DLB)		[20]
**VASCULAR DISEASE**		
Multiple vascular risk factors suggesting pathological processes associated with vascular dementia	-Presence of extensive cerebrovascular disease evident by structural MRI -“Step-wise” decline	[21]
**FRONTOTEMPORAL DEGENERATION**		
	-Frontal lobe atrophy evident by structural MRI -Decreased glucose metabolism in the frontal and temporal lobes evident by PET -Mutations in the microtubule-associatedprotein gene on chromosome 17	[22]

**Table 2:** Overview of different interventions in adults with MCI.

Intervention	Rationale	Key Message	References
Nutraceuticals	Targeted pathways include: -reducing oxidative stress and chronic inflammation -improving vascular function -supplementing macronutrients found in brain tissue and used in brain function	Few studies examined the effects of nutraceuticals on adults with MCI (i.e., omega-3, fatty acids, ginkgo biloba)	[24,25,26]
Hormone therapy	Speculation of the relationship between the pituitary endocrine axis and aging	-Low-strength evidence suggests that estrogen therapy may slightly increase the risk of probable MCI -Low-strength evidence suggests that faloxifene may decrease the risk of MCI compared to placebo -No effect of soybean-derived phosphatidylserine -Hormone therapy has been associated with serious adverse events, including increased risk of certain cancers and cardiovascular disease	[27,28]
Vitamin(s)	In the case of B vitamins the targeted pathway may involve lowering of homocysteine levels	-Moderate-strength evidence shows no benefit in cognitive performance for vitamin E in women -B vitamins show mixed findings -Low-strength evidence shows no benefit in cognitive performance for multivitamins, vitamin C (in women), vitamin D with calcium (in women), or -carotene (in women) -Low-strength evidence shows no benefit in incident MCI for multivitamins or vitamin D with calcium -In adults with MCI, low-strength evidence shows no benefit for vitamin E	[29,30,31,32,33]
Antihypertensive	Hypertension is thought to contribute to risk of both vascular and AD dementia through unclear vascular mechanisms. Presumably hypertension is the cause or result of vascular changes in the blood supply to the brain that can adversely affect its function. It remains unclear whether this is a direct effect or the result of other factors that affect both the vasculature and the brain.	Generally, low-strength evidence shows that 3 to 4.7 years of antihypertensive treatment regimens versus placebo appears to have no benefit on cognitive test performance in adults MCI	[34,35,36]
Lipid lowering treatment	Saturated fat intake is positively associated with MCI, or cognitive decline.	Evidence was insufficient to assess the effect of 5 years statin treatment on preventing MCI	[37]
Non-steroidal antiinflammatory drugs (NSAIDs)	Numerous epidemiological studies have shown an association between NSAID use and a reduced prevalence of dementia, specifically AD. In vitro and animal models of AD pathology show that NSAIDs reduce plaque-related inflammation and improve function, both at a cellular and behavioral level	No evidence is available for the effect of low-dose aspirin on MCI	[38,39]
Anti-dementia	The acetylcholinesterase inhibitors (AChEIs) have consistently demonstrated a modest but positive benefit to cognition in persons with mild through severe stages. They may likewise provide benefit to persons with age-related cognitive decline or MCI through the same mechanisms of action by increasing the duration of action of acetylcholine in the synapse through inhibition of its breakdown by AChE. The drugs have been approved by the FDA/EMA for people with mild to moderate AD but not for people with age-related cognitive decline or MCI.	-Low-strength evidence shows AChEIs do not reduce the incidence of AD in persons with MCI for over 3 years -Low-strength evidence shows AChEIs for 3 years have no significant effect on cognitive performance in adults with MCI	[33,40]
Diabetes medication	Diabetes may increase risk of AD through: -vascular mechanisms -direct effects of elevated blood glucose -insulin-resistance associated inflammation, and/or a pathway in which peripheral hyperinsulinemia inhibits brain insulin production, which then results in impaired brain Aclearance	No studies report on the effect of diabetes treatment on the risk of incident clinical diagnoses of MCI.	[41,42,43]
Other drugs		Evidence was insufficient for lithium, or for nicotine patch	[44,45]

Adapted from reference 23.

**Table 3:** Marine bioactive compounds acting on different targets shared by COPD and MCI/AD.

Drug	Mechanism		References
	COPD	MCI/AD	
**Activation PI3K-mTOR**			
Fucoxanthin	Inhibition of mice bleomicyn-induced lung fibrosis	Neuroprotective	[85,86]
Austrasulfone	NA	Anti-apoptotic	[87]
TMC-256C1	NA	Activation of kinases	[88]
1-*O*-(Myristoyl) glycerol (MG)	NA	Neurite outgrowth	[89]
Sargaquinoic acid	NA	Enhancement of neuriteregeneration	[90]
**Altered autophagy**			
Bafilomycins, coibamide A, manzamine A and papuamine	NA	Inhibits autophagy Induces autophagy Induces autophagy	[66]
**Defective DNA damage repair**			
Apo-9′-fucoxanthinone	Decreases cigarette smoke extract-induced DNA damage		[91]
**Cellular senescence**			
Antarctic krill oil (AKO)	NA	-Protective effect against AD senescence -Ameliorates learning and memory deficits and eases the anxiety -Reduces Aβ accumulation	[92]
Mitochondrial dysfunction			
43 kD protein	NA	-Attenuates neuronal cell death. -Alleviates mitochondrial inactivation -Recovers metallathionein-3 (MT-3) -Decreases and inhibits β- and γ-secretase, as well as Aβ accumulation	[93]
Gracilins	NA	-Positive trend on learning and spatial memory of treated mice. --Decreases Aβ42 and hyperphosphorylated levels -Preserves neurons against oxidative damage	[94,95]
